# Amplification and propagation of interleukin-1β signaling by murine brain endothelial and glial cells

**DOI:** 10.1186/s12974-017-0908-4

**Published:** 2017-07-01

**Authors:** Stephanie M. Krasnow, J. Gabriel Knoll, Santhosh Chakkaramakkil Verghese, Peter R. Levasseur, Daniel L. Marks

**Affiliations:** 1 Department of Pediatrics, Papé Family Pediatric Research Institute, Oregon Health & Science University, Portland, Oregon 97239 USA; 2 Oregon Health & Science University, Mail Code L481, 3181 SW Sam Jackson Park Rd., Portland, OR 97239 USA

**Keywords:** Interleukin-1β, Microglia, Astrocytes, Endothelium, Cytokines, NF-κB, Cell culture

## Abstract

**Background:**

During acute infections and chronic illnesses, the pro-inflammatory cytokine interleukin-1β (IL-1β) acts within the brain to elicit metabolic derangements and sickness behaviors. It is unknown which cells in the brain are the proximal targets for IL-1β with respect to the generation of these illness responses. We performed a series of in vitro experiments to (1) investigate which brain cell populations exhibit inflammatory responses to IL-1β and (2) examine the interactions between different IL-1β-responsive cell types in various co-culture combinations.

**Methods:**

We treated primary cultures of murine brain microvessel endothelial cells (BMEC), astrocytes, and microglia with PBS or IL-1β, and then performed qPCR to measure inflammatory gene expression or immunocytochemistry to evaluate nuclear factor kappa-light-chain-enhancer of activated B cells (NF-κB) activation. To evaluate whether astrocytes and/or BMEC propagate inflammatory signals to microglia, we exposed microglia to astrocyte-conditioned media and co-cultured endothelial cells and glia in transwells. Treatment groups were compared by Student’s *t* tests or by ANOVA followed by Bonferroni-corrected *t* tests.

**Results:**

IL-1β increased inflammatory gene expression and NF-κB activation in primary murine-mixed glia, enriched astrocyte, and BMEC cultures. Although IL-1β elicited minimal changes in inflammatory gene expression and did not induce the nuclear translocation of NF-κB in isolated microglia, these cells were more robustly activated by IL-1β when co-cultured with astrocytes and/or BMEC. We observed a polarized endothelial response to IL-1β, because the application of IL-1β to the abluminal endothelial surface produced a more complex microglial inflammatory response than that which occurred following luminal IL-1β exposure.

**Conclusions:**

Inflammatory signals are detected, amplified, and propagated through the CNS via a sequential and reverberating signaling cascade involving communication between brain endothelial cells and glia. We propose that the brain’s innate immune response differs depending upon which side of the blood-brain barrier the inflammatory stimulus arises, thus allowing the brain to respond differently to central vs. peripheral inflammatory insults.

**Electronic supplementary material:**

The online version of this article (doi:10.1186/s12974-017-0908-4) contains supplementary material, which is available to authorized users.

## Background

Infectious illnesses and chronic diseases elicit a constellation of metabolic and behavioral responses, including anorexia, weight loss, fever, lethargy, and disrupted sleep [1, 2]. These sickness responses evolved as part of a highly coordinated disease-fighting strategy and over a short term confer adaptive benefits by conserving energy and diverting it to the immune system. However, if the underlying illness does not resolve in a timely manner and these sickness responses persist, they become maladaptive and can lead to the development of cachexia, a wasting syndrome that develops in patients with chronic illnesses (e.g., cancer, AIDS, chronic obstructive pulmonary disease, congestive heart failure, and dementia) [3, 4].

Systemic inflammation is a common feature of the disparate infections and illnesses that cause sickness behaviors and cachexia [4]. Activated immune cells release pro-inflammatory cytokines that act upon target tissues in an autocrine, paracrine, or endocrine manner and play an integral role in coordinating the host’s immune response. The brain is one such target for pro-inflammatory cytokine signaling. In response to peripheral inflammatory insults, the hypothalamus generates its own local inflammatory response as a means to amplify and propagate the inflammatory signal within the central nervous system (CNS) [5]. This central inflammatory response involves many of the same cytokines that are released in the periphery (e.g., interleukin-1β (IL-1β), interleukin-6 (IL-6), and tumor necrosis factor-α (TNFα)) as well as chemokines that recruit leukocytes into the brain parenchyma (e.g., C-X-C motif chemokine 10 (CXCL10)). These inflammatory mediators modulate the activity of neural circuits controlling feeding, metabolism, body composition, arousal, and neuroendocrine function via direct and indirect mechanisms.

IL-1β plays a predominant role in centrally mediated sickness responses. Intracerebroventricular (icv) injection of IL-1β induces rapid and robust sickness behaviors in rodents [6–10], and blocking central IL-1β signaling attenuates sickness behaviors in response to peripheral injection of the bacterial endotoxin lipopolysaccharide (LPS) [11]. IL-1β signals through the type I interleukin-1 receptor (IL-1R1). In the rodent brain, *Il1r1* mRNA is primarily expressed by blood vessels, meninges, choroid plexus, and ependymal cells lining the cerebroventricles, but has also been reported in glia and discrete neuronal populations [12–16]. When IL-1β engages the IL-1R1, the adaptor protein myeloid differentiation factor 88 (MyD88) is recruited to the activated receptor complex. This triggers an intracellular signaling cascade that causes the transcription factor nuclear factor kappa-light-chain-enhancer of activated B cells (NF-κB) to translocate to the nucleus, where it binds to promoter regulatory elements and initiates transcription of inflammatory cytokine and chemokine genes [17]. Although most IL-1β-induced inflammatory genes are regulated by NF-κB signaling, IL-1β can also activate MAPK pathways [18]. MyD88 is requisite for many pro-inflammatory actions of IL-1β in the CNS, but IL-1β can signal via a MyD88-independent pathway in hypothalamic neurons [19].

It is unknown which cell population(s) in the brain is/are the proximal targets for IL-1β with respect to the generation of sickness responses. MyD88 knockout (MyD88KO) mice are resistant to IL-1β-induced sickness behaviors [6, 20]. Although populations of hypothalamic neurons that regulate feeding and metabolism express IL-1R1 and are activated or inhibited by IL-1β [14, 15], these neurons do not appear to be the exclusive targets for IL-1β-induced sickness behaviors, because mice in which MyD88 is selectively deleted from neurons and astrocytes exhibit normal sickness behaviors in response to icv IL-1β [6]. In contrast, conditional deletion of MyD88 from endothelial and myeloid cells (including microglia) driven by the Tie2 promoter confers resistance to anorexia, weight loss, reduced locomotor activity, and fever in response to icv IL-1β [8].

The goal of these experiments was to examine the inflammatory responses of endothelial cells, microglia and astrocytes to IL-1β. While others have previously reported the effects of IL-1β on cellular activation and inflammatory gene expression in vivo and in isolated brain cell populations in vitro, less effort has been devoted to examining the interactions between different IL-1β-responsive brain cell populations, the directionality of signaling, or the potential for synergistic cellular actions. To this end, we took a systematic in vitro approach and measured inflammatory gene expression and NF-κB activity in primary mouse brain endothelial and glial cells, as well as in a recently described spontaneously transformed murine microglia cell line (SIM-A9) [21]. We demonstrate that in response to IL-1β, microglia exhibit minimal inflammatory responses in isolation, but generate more robust responses when co-cultured with astrocytes and/or endothelial cells. We also find that the endothelial response to IL-1β stimulation is polarized, because application of IL-1β to the abluminal endothelial surface produces a more complex microglial response than that which occurs after the luminal endothelial membrane is exposed to IL-1β.

## Methods

### Animals

Adult male and female C57BL/6J (wild-type; WT), MyD88 knockout (MyD88KO), and CX3CR1-EYFP-Cre mice were purchased from the Jackson Laboratory (Bar Harbor, ME). Mice were housed in a light- and temperature-controlled room and were provided with food and water ad libitum. All experiments were conducted in accordance with the National Institutes of Health Guide for the Care and Use of Laboratory Animals and were approved by the Animal Care and Use Committee of Oregon Health & Science University.

### Drugs

Murine IL-1β (R&D Systems, Minneapolis, MN), murine TNF-α (R&D Systems), and l-leucine methyl ester hydrochloride (l-LME; Sigma, St. Louis, MO) were dissolved in PBS. LPS (Sigma) was dissolved in PBS + 0.1% bovine serum albumin (BSA). Nω-Nitro-l-arginine methyl ester hydrochloride (l-NAME; Sigma) was dissolved in phenol red-free DMEM (#31053, Life Technologies, Carlsbad, CA) supplemented with l-glutamine (2 mM) and gentamicin (50 μg/mL).

### Primary brain microvessel endothelial cell cultures

Primary brain microvessel endothelial cell (BMEC) cultures were generated as previously described, with a few modifications [22]. For each culture, 5 to 10 adult WT mice were decapitated under isoflurane anesthesia. Forebrains were isolated and separated from the meninges, minced, and then transferred to a tube containing collagenase CLS2 (1 mg/mL; Worthington, Lakewood, NJ) and DNase I (10 μg/mL; Sigma) in ggDMEM [DMEM (#11965) supplemented with l-glutamine (2 mM) and gentamicin (50 μg/mL)] for 45 min at 37 °C in a shaking water bath. Cells were resuspended in endothelial culture medium containing 20% BSA (pH 7.4) and were spun at 1000×*g* for 20 min at 4 °C. After aspirating the myelin-rich layer and supernatant, the cells were mixed with collagenase/dispase (1 mg/mL; Sigma) and DNase I (10 μg/mL) in ggDMEM and were incubated in a shaking 37 °C water bath for 30 min. Cells were then layered on a 33% Percoll (GE Healthcare Life Sciences, Pittsburgh, PA) gradient and spun at 1000×*g* for 20 min at 4 °C. The microvessel layer was removed, mixed with ggDMEM, and spun for 8 min at 700×*g*. At the end of the isolation, pellets were resuspended in endothelial culture medium [ggDMEM + endothelial cell growth supplement (100 μg/mL; Sigma) + 20% fetal bovine serum (FBS; Hyclone, Logan, UT)) + heparin (100 μg/mL; Sigma)] supplemented with puromycin (4 μg/mL; Sigma). Seventy-two h after seeding fragments into collagen-coated flasks, cells were washed with PBS and switched to puromycin-free culture medium. Medium was changed every 2–3 days until the cells reached confluence. Cells were then trypsinized and re-plated into 6-well plates for RNA analysis or 24-well plates containing poly-d-lysine/laminin-coated coverslips (Corning, Corning, NY) for immunocytochemistry (ICC).

### Primary mixed glia cultures

The brains were harvested from neonatal mouse pups on P1-P5. Cortices were dissected, separated from the meninges, rinsed in dissecting buffer (350 mg/L NaHCO_3_, 6 g/L d-glucose, 300 mg/L BSA, 1.44 g/L MgSO_4_*7H_2_O, and 10 mM HEPES in Hank’s Balanced Salt Solution) and digested with papain (Worthington) for 5 min. The enzymatic digestion was terminated by the addition of culture medium [DMEM (#11885; Life Technologies) + 10% FBS + 1% penicillin/streptomycin (Life Technologies)]. Cell suspensions were filtered and spun at 150×*g* for 10 min. Cells were seeded into T-75 flasks and placed in a 37 °C incubator with 5% CO_2_/95% O_2_. Culture medium was changed 24 h later, and then every 3–5 days thereafter. Between days in vitro (DIV)10–DIV20, mixed glia cultures were dissociated with 0.05% trypsin-EDTA and seeded into 6-well plates (for RNA analysis), 24-well plates containing poly-d-lysine/laminin-coated coverslips for ICC, or the upper inserts of transwells for luciferase assays.

### Astrocyte-enriched cultures

Neonatal mouse cortices were processed as described above, and cells were seeded into 6-well (1.5 × 10^6^ cells/well) or 24-well (1.5 × 10^5^ cells/well) plates. Seventy-two h later, 1 mM l-LME was added to each well to selectively deplete microglia [23]. l-LME treatment was repeated again 3 days later on DIV6. On DIV9, astrocytes were treated with 0.05% trypsin-EDTA, spun at 150×*g* for 10 min, and then seeded into 6-well plates for RNA analysis.

### Microglia isolation

Microglia-enriched cultures were derived from mixed glia cultures using a mild trypsinization technique [24]. Compared to the more commonly used shaking method of isolating microglia, mild trypsinization has been reported to generate higher and purer yields of microglia, and the isolated microglia are less activated at baseline [25]. Between DIV16 and DIV19, mixed glia cultures were incubated in 0.25% trypsin-EDTA diluted 1:3 in serum-free DMEM for 30 min at 37 °C, and then the detached astrocyte layer was aspirated. The underlying adherent microglia were dissociated with 0.25% trypsin-EDTA and seeded into 24-well plates containing poly-d-lysine/laminin-coated coverslips for ICC, or were returned to the incubator for subsequent gene expression analyses.

### SIM-A9 microglia cell line

SIM-A9 cells were kindly provided by Dr. Kumi Nagamoto-Combs [21]. DNA was extracted with a DNeasy kit (Qiagen, Valencia, CA), and the SRY gene was amplified by PCR (forward primer: 5′-AGGCGCCCCATGAATGCATT-3′; reverse primer: 5′-TCCGATGAGGCTGATATTTATAG-3′). The lack of a band in SIM-A9 cells revealed that these cells are of female origin (data not shown). Cells were grown in DME/F-12 (Hyclone, #SH-30023) + 10% FBS + 10% donor horse serum (Serum Source International, Charlotte, NC) + 1% penicillin/streptomycin. Cells were detached with splitting medium (1 mM EDTA, 1 mM EGTA, and 1 mg/mL glucose in PBS) and seeded into 6- or 12-well plates.

### NF-kB luciferase SIM-A9 cells

Human embryonic kidney cells (HEK293T) were seeded at a density of 1.5 × 10^7^ cells/15-cm tissue culture dish (Corning) after coating the dishes with 0.01% poly-l-lysine (Sigma). The HIV-1-based lentiviral vector stocks were produced by co-transfecting the helper constructs pLP1, pLP2, and pLP/VSVG [26] and the transducing plasmid pHAGE NFkB-TA-LUC-UBC-GFP-W (a gift from Darrell Kotton (Addgene Plasmid #49343)) [27]. SIM-A9 cells were transduced with the harvested vector pHAGE NFkB-TA-LUC-UBC-GFP-W in the presence of 8 μg/ml protamine sulfate (MP Biomedicals, Santa Ana, CA) and incubated at 37° C overnight [28]. Cells were washed, spun for 5 min at 1000 rpm, and used for FACS analysis followed by sorting the GFP-positive vector transduced cells (Canto-II, BD InFlux cell sorter; BD Biosciences, San Jose, CA). Sorted cells were washed twice prior to expansion.

### Transwell BMEC experiments

Primary BMEC were isolated as described above, with a few deviations. After spinning the endothelial fragments in the Percoll gradient, cells were resuspended in culture medium containing endothelial cell growth supplement (60 μg/mL), 20% plasma-derived platelet-free serum (Alfa Aesar, Ward Hill, MA), heparin (100 μg/mL), Glutamax (2 mM, Life Technologies), and puromycin (4 μg/mL). Endothelial fragments were seeded onto collagen-coated transwell inserts (catalog #3460, Corning Costar, Corning, NY). After 72 h, BMEC were switched to puromycin-free culture medium and were co-cultured with WT primary mixed glia in the lower wells. BMEC were confluent after 4 days in co-culture with mixed glia, at which time the transwell inserts were moved to wells containing SIM-A9 or NF-kB Luc SIM-A9 cells. We used SIM-A9 cells as surrogates for primary microglia because we had difficulty isolating the large numbers of primary microglia that would be necessary for these experiments. BMEC were treated with PBS or IL-1β (50 ng/mL) added to either the luminal surface (upper chamber) or abluminal surface (lower chamber) for 6 or 8 h. Cells were lysed and then assayed for luciferase activity or frozen at −80 °C for gene expression analysis. Transwell inserts were pooled (two inserts pooled together) for measuring BMEC gene expression. In a separate experiment, once the BMEC reached confluence, the transwell inserts were moved to wells that contained CX3CR1-EYFP-Cre primary mixed glia on poly-d-lysine/laminin-coated coverslips. PBS or IL-1β (50 ng/mL) was added to both the upper and lower chambers for 30 min before proceeding with immunostaining.

### Nitric oxide synthesis blockade

BMEC and SIM-A9 cells were seeded into transwells as described above. Cells were switched to phenol red-free DMEM supplemented with 2 mM l-glutamine and 50 μg/mL gentamicin (phenol red-free ggDMEM). l-NAME (1 mM) or phenol red-free ggDMEM was added to both the upper and lower chambers for 1 h. Then, PBS or IL-1β (50 ng/mL) was added to the upper chambers (i.e., the luminal endothelial surface) for an additional 8 h. SIM-A9 cells were lysed and frozen at −80 °C for gene expression analysis. Supernatants from the upper chambers were analyzed for nitrate/nitrite levels using a nitrate/nitrite colorimetric assay kit (Cayman Chemical, Ann Arbor, MI) following the manufacturer’s instructions.

### Conditioned media experiments

WT astrocyte-enriched cultures were treated with PBS or IL-1β (50 ng/mL) for 24 h, after which the astrocyte-conditioned media was removed and added to wells containing WT primary microglia or SIM-A9 cells for an additional 4 h. Gene expression was measured in the astrocytes, primary microglia, and SIM-A9 cells.

### Gene expression analyses

All gene expression studies were conducted 24–48 h after cells were seeded into 6- or 12-well plates. Cells were washed with PBS and then switched to serum-free media for 30 min. Cells were then treated with vehicle (PBS or PBS + 0.1% BSA), IL-1β (50 ng/mL), TNF-α (50 ng/mL), or LPS (10 ng/mL) for 4 h prior to lysis and stored at −80 °C. SIM-A9 cells were treated with PBS or IL-1β (50 ng/mL) for 1, 2, 4, 8, or 24 h. Total cellular RNA was extracted using RNeasy kits (Qiagen). cDNA was generated using Taqman reverse transcription reagents as previously described [29]. Real-time PCR was performed with Taqman reagents using an ABI 7300 system (Life Technologies). Each sample was run in triplicate, with 18S or β-actin as endogenous controls. Gene expression is presented in terms of relative quantity, or foldchange relative to the vehicle (control) group, and was calculated using the 2^−ΔΔCt^ method. Statistical analyses were performed on the ΔC_t_ values for each gene.

### Luciferase assay

NF-kB Luc SIM-A9 cells were seeded into empty 12-well or 48-well plates, 12-well plates containing WT primary mixed glia, or in the lower chambers of transwell plates with WT primary mixed glia in the upper chambers. Forty-eight h later, cells were washed in PBS and incubated in serum-free media for 30 min prior to treatment. Cells were treated with vehicle (PBS or PBS + 0.1% BSA), IL-1β (50 or 100 ng/mL), or LPS (10 or 100 ng/mL) for 6 h and then lysed in Glo Lysis buffer (Promega Corporation, Madison, WI). Luciferase activity in the lysates was measured using the Bright-Glo Luciferase Assay System (Promega) and a BioTek Gen5 microplate reader (Winooski, VT). Luminescence was normalized to total protein content, which was measured using a Pierce BCA protein assay kit (Thermo Fisher Scientific, Waltham, MA) according to the manufacturer’s instructions.

### Measurement of supernatant IL-1β

WT BMEC were seeded into the upper inserts of 16 transwells. In eight of the transwells, SIM-A9 cells were seeded into the lower chambers. The other eight transwells contained empty lower chambers. PBS or IL-1β (50 ng/mL) was added to the upper chambers (*n* = 4 each for SIM-A9 + PBS, SIM-A9 + IL-1β, empty lower chambers + PBS, and empty lower chambers + IL-1β). After 8 h, the supernatant was removed from both the upper and lower chambers and frozen at −80 °C. Supernatant IL-1β was measured by ELISA (Thermo Fisher Scientific, Mouse IL1β ELISA Ready SET Go) according to the manufacturer’s instructions. Sensitivity of the assay was 8 pg/mL.

### ICC

Mixed glia, primary microglia, and BMEC were processed for ICC 24–48 h after they were seeded onto poly-d-lysine/laminin-coated coverslips. Cells were washed in PBS and incubated in serum-free media for 30 min. Cells were then treated with vehicle (PBS or PBS + 0.1% BSA), IL-1β (50 ng/mL), or LPS (10 ng/mL) for 30 min. Cells were then fixed in 4% paraformaldehyde for 30 min and blocked for 30 min in 0.3% Triton-X 100 + 1% BSA in PBS. Cells were incubated with primary antibodies diluted in PBS + 0.3% Triton-X 100 + 5% normal serum at 4 °C overnight. Primary antibodies were used at the following concentrations: rabbit anti-p65 NF-κB (1:1000; Cell Signaling, Danvers, MA), mouse anti-GFAP (1:2000; Millipore, Billerica, MA), rat anti-PE-CAM (1:100; BD Pharmingen, San Jose, CA), and chicken anti-GFP (1:1000; Abcam, Cambridge, MA). The next day, cells were incubated in Alexa Fluor secondary antibody (diluted in PBS + 0.3% Triton-X 100 + 1% normal serum) for 2 h at room temperature. The secondary antibodies were donkey anti-rabbit 555, goat anti-mouse 633, donkey anti-rat 488, and goat anti-chicken 488 (Life Technologies). Cells were stained with DAPI and mounted onto gelatin-coated slides using Aqua-Poly/Mount (PolySciences, Inc., Warrington, PA). Cells were imaged with a Nikon Ti Eclipse inverted microscope and NIS Elements software (Nikon Instruments Inc, Melville, NY). Grayscale images were merged and pseudo-colored using Adobe Photoshop CS6 (Adobe Systems, San Jose, CA). To quantify microglial NF-κB nuclear localization, 10 random fields from each coverslip (2–3 coverslips per treatment group) were imaged. An observer who was blinded to the treatment groups tabulated the percentage of YFP-positive cells with concentrated nuclear NF-kB labeling. To quantify BMEC NF-κB nuclear localization, three random fields from each coverslip or transwell (two to three coverslips or transwells per treatment group) were imaged. The percentage of DAPI-labeled nuclei that were co-labeled with NF-κB was tabulated by a blinded observer. For occludin immunostaining, transwell inserts containing BMEC were fixed in ice cold 100% ethanol for 30 min, blocked for 30 min in PBS + 3% FBS, and then incubated in rabbit anti-occludin antibody (Thermo Fisher Scientific; 1:200) at 4 °C overnight. The next day, cells were incubated in goat anti-rabbit 488 (Life Technologies) for 2 h at room temperature and then mounted with Fluoromount-G with DAPI. Cells were imaged with a DM4000 B fluorescent microscope (Leica Microsystems, Buffalo Grove, IL) equipped with a DFC340 FX camera (Leica).

### Statistical analysis

Data are expressed as the mean ± SEM for each group. Statistical analyses were performed using GraphPad Prism 5 for Mac OS X. Groups were compared by Student’s *t* tests or ANOVA followed by Bonferroni-corrected *t* tests. For the SIM-A9 time course experiment, groups were compared by two way ANOVA (time × treatment) followed by Bonferroni-corrected *t* tests. Differences were considered significant when *p* < 0.05.

## Results

### Baseline Il1r1 and Myd88 mRNA expression

We measured relative basal *Il1r1* and *Myd88* gene expression in BMEC, mixed glia, enriched astrocytes, and primary microglia that were harvested from WT mice and in SIM-A9 cells (Table 1). *Il1r1* expression was similar in BMEC and enriched astrocytes, slightly lower in mixed glia cultures, and lowest in primary microglia. *Il1r1* was undetectable in SIM-A9 cells. In contrast, *Myd88* mRNA expression was highest in primary microglia, lower in SIM-A9 cells and enriched astrocytes, and lowest in BMEC and mixed glia.

**Table 1:** Baseline *Il1r1* and *Myd88* mRNA expression

	*Il1r1* ΔC_t_	*Myd88* ΔC_t_
BMEC	14.05 ± 0.10	15.57 ± 0.07
Mixed glia	16.64 ± 0.19	16.39 ± 0.11
Enriched astrocytes	14.83 ± 0.08	15.08 ± 0.07
Primary microglia	21.50 ± 0.27	13.55 ± 0.09
SIM-A9	Not detected	14.71 ± 0.06

*Il1r1* ΔC_t_ = *Il1r1* C_t_– *18S* C_t_; *Myd88* ΔC_t_ = *Myd88* C_t_– *18S* C_t_. *n* = 3 to *n* = 7 per group

### BMEC inflammatory gene expression and NF-kB activity in response to IL-1β

**Fig. 1:**
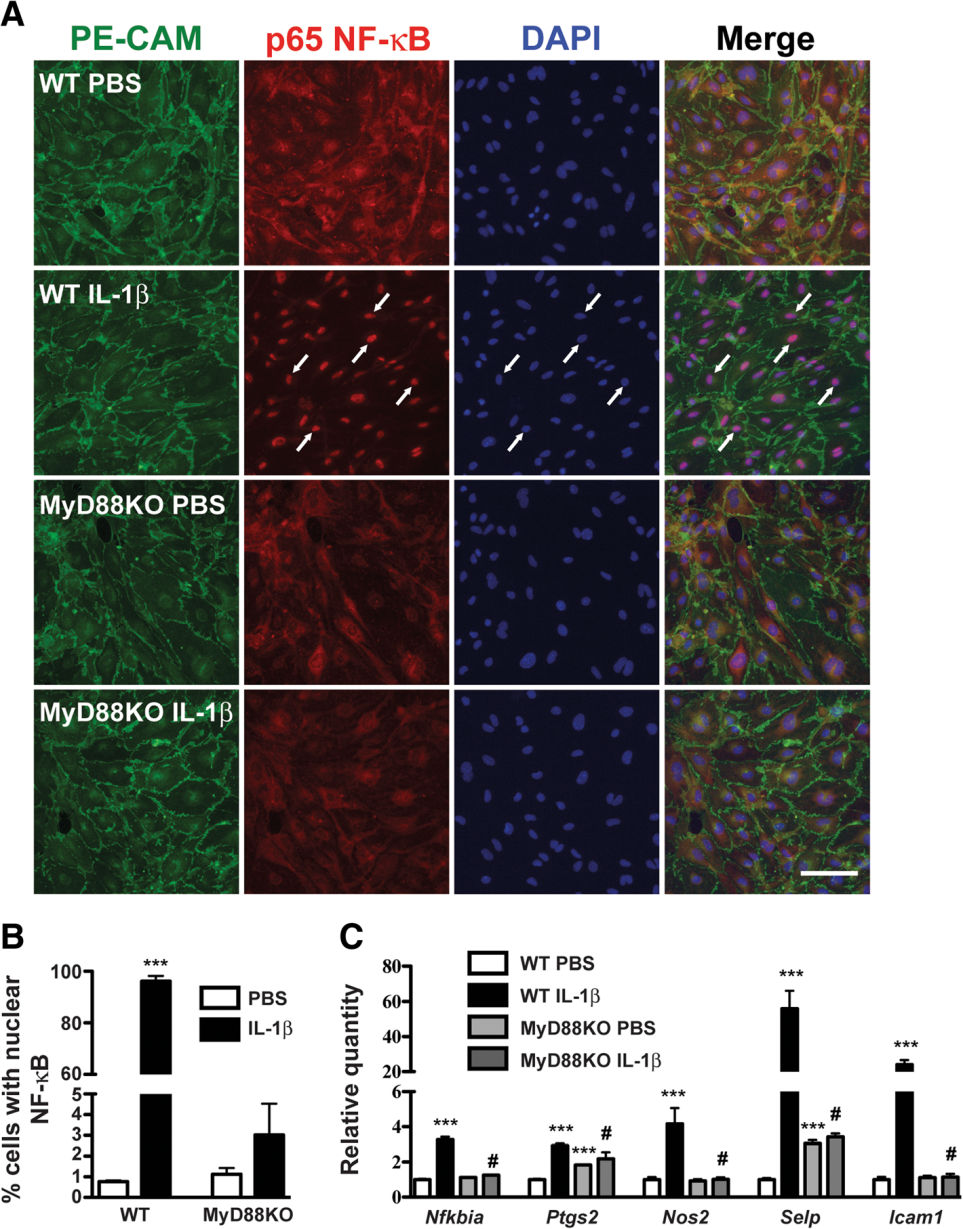
Brain microvessel endothelial cells (BMEC) respond to IL-1β in a MyD88-dependent manner. **a** PE-CAM (*green*) and p65 NF-kB (*red*) immunofluorescence in WT and MyD88KO BMEC that were treated with PBS or IL-1β (50 ng/mL) for 30 min. Blue = DAPI. *Arrows* indicate examples of PE-CAM-positive cells with nuclear concentration of NF-κB. Scale bar = 50 μm. **b** Percentage of BMEC with nuclear concentration of NF-κB. **c** Inflammatory gene expression in WT and MyD88KO BMEC that were treated with PBS or IL-1β (50 ng/mL) for 4 h. *n* = 2 to *n* = 3 per group. Data are expressed as mean ± SEM. ***p < 0.001 vs. WT PBS group, #*p* < 0.05 vs. WT IL-1β group

We investigated the effects of IL-1β on primary BMEC cultures and observed nuclear translocation of immunoreactive NF-κB in IL-1β-treated BMEC harvested from WT mice, but not in MyD88KO BMEC (Fig. 1a and b). IL-1β also increased gene expression of inhibitor of kappa B alpha (Iκ-Bα, a transcriptional marker of NF-κB activity that is encoded by the *Nfkbia* gene) in cultured BMEC from WT mice, but not in MyD88KO endothelial cells (Fig. 1c). Brain endothelial cells produce prostaglandins and nitric oxide in response to inflammatory stimuli. After 4 h, IL-1β increased the expression of the prostaglandin synthetic enzyme cyclooxygenase-2 (*Ptgs2*) and inducible nitric oxide synthase (*Nos2*) in WT but not MyD88KO BMEC (Fig. 1c). BMEC also express the cell adhesion molecules intercellular adhesion molecule 1 (encoded by the *Icam1* gene) and P-selectin (encoded by the *Selp* gene), both of which promote leukocyte extravasation. We observed large increases in *Icam1* (24-fold) and *Selp* (56-fold) mRNAs in WT but not MyD88KO endothelial cells in response to IL-1β (Fig. 1c). Collectively, these data demonstrate that brain endothelial cells respond to IL-1β in a MyD88-dependent manner.

### Astrocyte and mixed glia transcriptional responses to IL-1β

IL-1β upregulated *Nfkbia*, cytokine (*Il1b*, *Il6* and *Tnf*), *Cxcl10*, *Nos2*, and *Ptgs2* mRNA expression in mixed glia and astrocyte-enriched cultures derived from WT mice (Additional file 1). Although the pattern of inflammatory gene expression in astrocytes mirrored the mixed glia response to IL-1β, the increases in gene expression in enriched astrocyte cultures were less robust than the corresponding mixed glia responses. This was especially true for *Nos2*, which was induced by an order of magnitude more in mixed glia than in enriched astrocytes. We did not observe any IL-1β-induced changes in inflammatory gene expression in MyD88KO mixed glia or enriched astrocyte cultures.

### Primary microglia and SIM-A9 responses to inflammatory stimuli

**Fig. 2:**
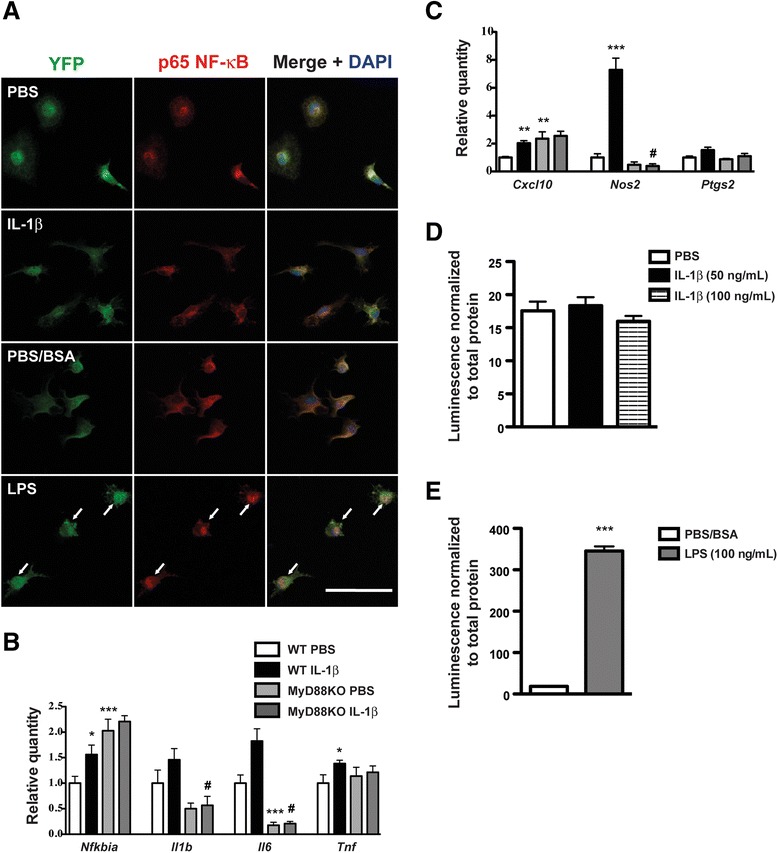
Microglia exhibit small transcriptional responses to IL-1β. **a** Representative images of NF-κB immunofluorescence in CX3CR1-YFP-Cre primary microglia that were treated with PBS, IL-1β (50 ng/mL), PBS + 0.1% BSA, or LPS (10 ng/mL) for 30 min. *Green* = YFP, *red* = p65 NF-kB, *blue* = DAPI. Scale bar *=* 50 μm. *Arrows* identify cells with nuclear NF-κB. **b**, **c** Inflammatory gene expression in primary microglia from WT and MyD88KO mice that were treated with PBS or IL-1β (50 ng/mL) for 4 h. Data are representative of two independent experiments. *n* = 4 to *n* = 7 per group. Data are expressed as mean ± SEM. *p < 0.05, **p < 0.01, ***p < 0.001 vs. WT PBS, #*p* < 0.05 vs. WT IL-1β. **d**, **e**. Luciferase activity in NF-κB Luc SIM-A9 cells that were treated with PBS, IL-1β (50 or 100 ng/mL), PBS + 0.1% BSA, or LPS (100 ng/mL) for 6 h. Luminescence was normalized to total protein content. Data from two independent experiments were combined. *n* = 7 to *n* = 12 per group. Data are expressed as mean ± SEM. ***p < 0.001 vs. PBS/BSA group

In primary cortical microglia cultures obtained from CX3CR1-EYFP-Cre mice (which have constitutive microglial EYFP expression), we observed nuclear NF-κB in virtually all the cells after LPS treatment, but only rarely in microglia that were treated with IL-1β (Fig. 2a). *Nfkbia* gene expression was modestly (1.6-fold; *p* < 0.05 vs. PBS WT group) increased by IL-1β treatment in WT but not MyD88KO microglia (Fig. 2b). Expression of *Il1b*, *Il6*, and *Ptgs2* mRNAs was not altered by IL-1β in WT or MyD88KO microglia (Fig. 2b, c). In WT microglia, IL-1β induced small but statistically significant increases in *Tnf* (1.4-fold) and *Cxcl10* (2.0-fold) mRNAs. *Nos2* was the only gene that was highly upregulated (7.8-fold) by IL-1β in WT microglia. Similarly, IL-1β elicited small but statistically significant increases in the expression of inflammatory genes in the microglial cell line SIM-A9 (Additional file 2). Whereas *Tnf*, *Nfkbia*, and *Cxcl10* mRNAs were only transiently increased by IL-1β, *Il1b* gene expression was induced as early as 2 h following IL-1β treatment and remained elevated at the 24 h time point. Although *Nos2* mRNA expression also increased following IL-1β treatment, the differences did not reach statistical significance at any time point. We also measured luciferase activity in SIM-A9 cells that were stably transduced with an inducible NF-κB luciferase construct (NF-κB Luc SIM-A9 cells), but we did not detect increased luminescence in response to IL-1β (Fig. 2d).

To confirm that primary microglia and SIM-A9 cells are capable of exhibiting robust responses to inflammatory stimuli, we treated these cells with LPS (which also signals via MyD88) or TNFα (a MyD88-independent cytokine). Primary microglia and SIM-A9 cells exhibited pronounced increases in *Il1b*, *Il6*, *Tnf*, *Nfkbia*, *Cxcl10*, *Nos2*, and *Ptgs2* gene expression in response to LPS (Additional file 3), and LPS increased luciferase activity in NF-κB Luc SIM-A9 cells (Fig. 2e). TNFα also induced expression of the same inflammatory genes in SIM-A9 cells after 4 h of treatment (Additional file 3). Thus, although primary cortical microglia and SIM-A9 cells exhibit robust transcriptional responses to MyD88-dependent and MyD88-independent inflammatory signaling molecules, these cells demonstrate only subtle changes in inflammatory gene expression in response to IL-1β.

### IL-1β-induced astrocyte-to-microglia communication

**Fig. 3:**
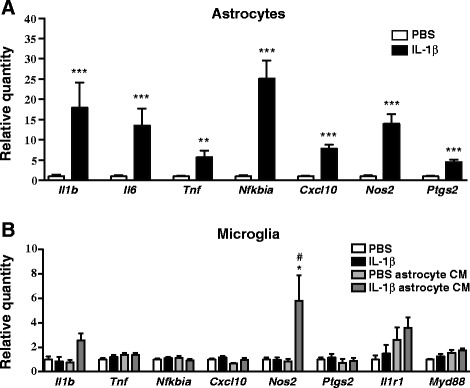
Effect of IL-1β-treated astrocyte-conditioned media (CM) on microglia gene expression. **a** Inflammatory gene expression in enriched astrocyte cultures that were treated with PBS or IL-1β (50 ng/mL) for 24 h. *n* = 5 per group. Data are expressed as mean ± SEM. **p < 0.01, ***p < 0.001 vs. PBS group. **b** Inflammatory gene expression in primary WT microglia that were treated with PBS-treated astrocyte CM (PBS astrocyte CM) or IL-1β-treated astrocyte CM (IL-1β astrocyte CM) for 4 h. To control for direct effects of IL-1β on primary microglia, empty wells (containing no astrocytes) were treated with PBS or IL-1β for 24 h, and then the media was removed and applied to primary microglia for 4 h. These microglia treatment groups are labeled as PBS and IL-1β, respectively. *n* = 3 to *n* = 4 per group. Data are expressed as mean ± SEM. *p < 0.05 vs. PBS astrocyte CM group, #*p* < 0.05 vs. IL-1β group

**Fig. 4:**
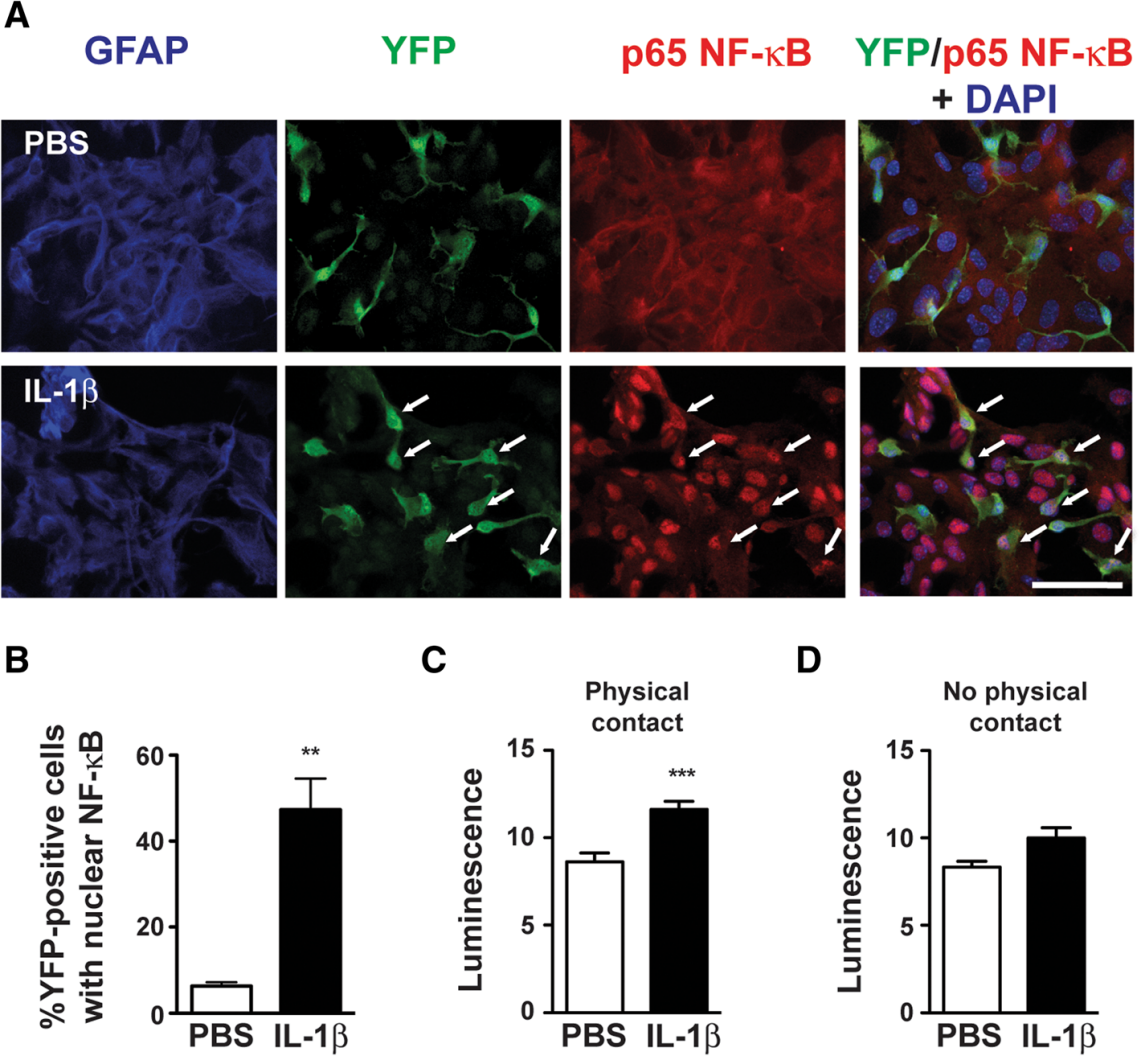
NF-kB activity in IL-1β-treated microglia and astrocytes. **a** GFAP (*blue*), YFP (*green*), and p65 NF-kB (*red*) immunofluorescence in primary mixed glia cultures from CX3CR1-EYFP-Cre mice that were treated with PBS or IL-1β (50 ng/mL) for 30 min. In the far right column, DAPI labeling is shown in *blue*. Scale bar *=* 50 μm. *Arrows* indicate YFP-positive microglia with concentrated nuclear NF-κB expression. **b** Percentage of YFP-positive cells with concentrated nuclear NF-κB. **c** Luminescence in NF-κB Luc SIM-A9 cells that were co-cultured with WT mixed glia and treated with PBS or IL-1β (50 ng/mL) for 6 h. Data from two independent experiments were combined. *n* = 8 per group. **d** Luminescence in NF-κB Luc SIM-A9 cells that were seeded into the lower chambers of transwell plates containing WT mixed glia in the upper inserts. The mixed glia were treated with PBS or IL-1β (50 ng/mL) for 6 h. *n* = 3 per group. Data are expressed as mean ± SEM. **p < 0.01, ***p < 0.001 vs. PBS group

Having established that astrocytes exhibit more pronounced inflammatory responses to IL-1β than microglia, we next investigated whether microglia respond to paracrine signals released by IL-1β-treated astrocytes. First, we exposed WT astrocyte-enriched cultures to IL-1β for 24 h, and then treated primary microglia or SIM-A9 cells with the astrocyte-conditioned media for an additional 4 h. Astrocytes responded to IL-1β with significant increases in *Il1b*, *Il6*, *Tnf*, *Nfkbia*, *Cxcl10*, *Ptgs2*, and *Nos2* gene expression (Fig. 3a and Additional file 4A). Treating primary microglia with conditioned media from IL-1β-treated astrocytes significantly increased *Nos2* mRNA (6.9-fold compared to conditioned media from PBS-treated astrocytes, Fig. 3b). There were no significant effects on gene expression in SIM-A9 cells that received conditioned media from IL-1β-treated astrocytes, compared to SIM-A9 cells that received conditioned media from PBS-treated astrocytes (Additional file 4B). Next, we examined whether IL-1β induces nuclear NF-κB localization in primary cultures comprised of both microglia and astrocytes. After 30 min of IL-1β treatment, we observed nuclear NF-κB in most astrocytes and in approximately half of the microglia (Fig. 4a, b). We also detected increased luminescence in NF-κB Luc SIM-A9 cells that were co-cultured with WT mixed glia and then treated with IL-1β, thereby providing further evidence for IL-1β-induced microglial NF-κB activation when astrocytes are also present in the culture dish (Fig. 4c). In contrast, we did not observe increased luminescence when NF-κB Luc SIM-A9 cells were seeded into the lower chambers of transwells with IL-1β-treated wild-type mixed glia in the upper inserts (Fig. 4d).

### BMEC-to-microglia communication in response to IL-1β

**Fig. 5:**
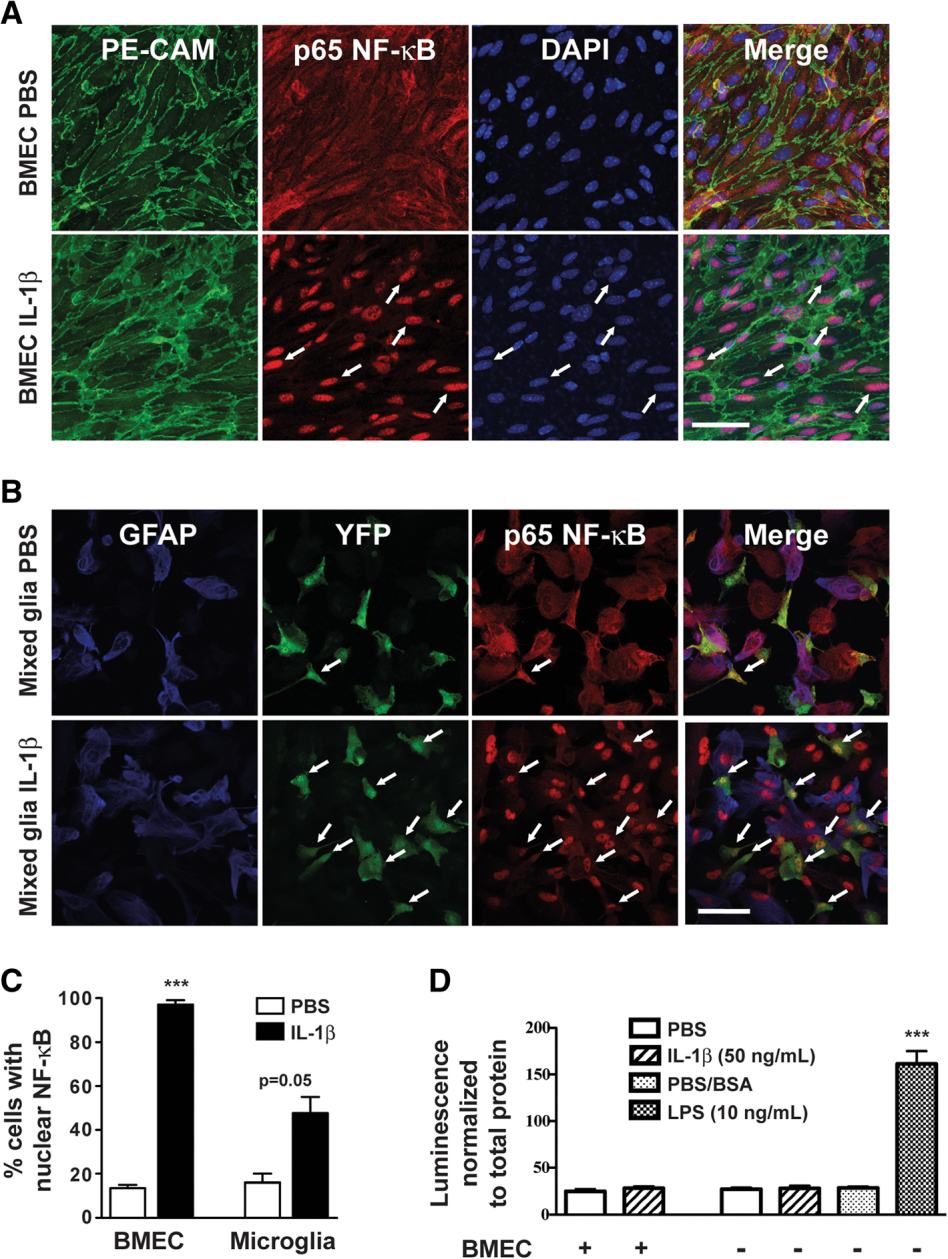
NF-κB localization in co-cultured BMEC, astrocytes, and microglia. **a** PE-CAM (*green*) and p65 NF-kB (*red*) immunofluorescence in WT BMEC that were seeded into transwell inserts and treated with PBS or IL-1β (50 ng/mL) for 30 min. *Blue* = DAPI. Scale bar *=* 50 μm. *Arrows* indicate examples of PE-CAM-positive cells with concentrated nuclear NF-κB expression. **b** GFAP (*blue*), YFP (*green*), and p65 NF-κB (*red*) immunofluorescence in CX3CR1-YFP-Cre mixed glia that were seeded into lower transwell chambers containing PBS- or IL-1β-treated BMEC in the upper inserts. Scale bar *=* 50 μm. *Arrows* indicate nuclear expression of NF-κB in YFP-labeled cells. **c** Percentage of BMEC and microglia with nuclear NF-κB localization. ***p < 0.001 vs. PBS-treated BMEC. **d** Luciferase activity in NF-κB Luc SIM-A9 cells that were co-cultured with WT BMEC in transwells. PBS or IL-1β (50 ng/mL) was added to both the upper and lower chambers for 6 h. NF-κB Luc SIM-A9 cells were also treated with PBS, IL-1β (50 ng/mL), PBS + 0.1% BSA, or LPS (10 ng/mL) for 6 h in the absence of BMEC. Luminescence was normalized to total protein content. *n* = 4 per group. Data are expressed as mean ± SEM. ***p < 0.001 vs. all other groups

We next examined whether IL-1β activates microglia when they are co-cultured with BMEC. WT BMEC were seeded into the upper chambers of transwell plates, and CX3CR1-EYFP-Cre mixed glia cultures were placed in the lower chambers. Both upper and lower chambers were treated with PBS or IL-1β for 30 min. NF-κB was diffusely localized throughout the cytosol in the PBS-treated BMEC, as well as in the co-cultured astrocytes and microglia (Fig. 5a, b). Following IL-1β treatment, NF-κB was observed in the nuclei of virtually all BMEC and astrocytes, as well as in approximately half of the microglia (Fig. 5c). Next, we seeded NF-κB Luc SIM-A9 cells into the lower chambers of transwell plates that contained WT BMEC in the upper chambers. Addition of IL-1β to both chambers for 6 h significantly increased *Il6*, *Nos2*, and *Ptgs2* mRNA expression in the BMEC (data not shown), but did not increase luminescence in the underlying NF-κB Luc SIM-A9 cells (Fig. 5d).

**Fig. 6:**
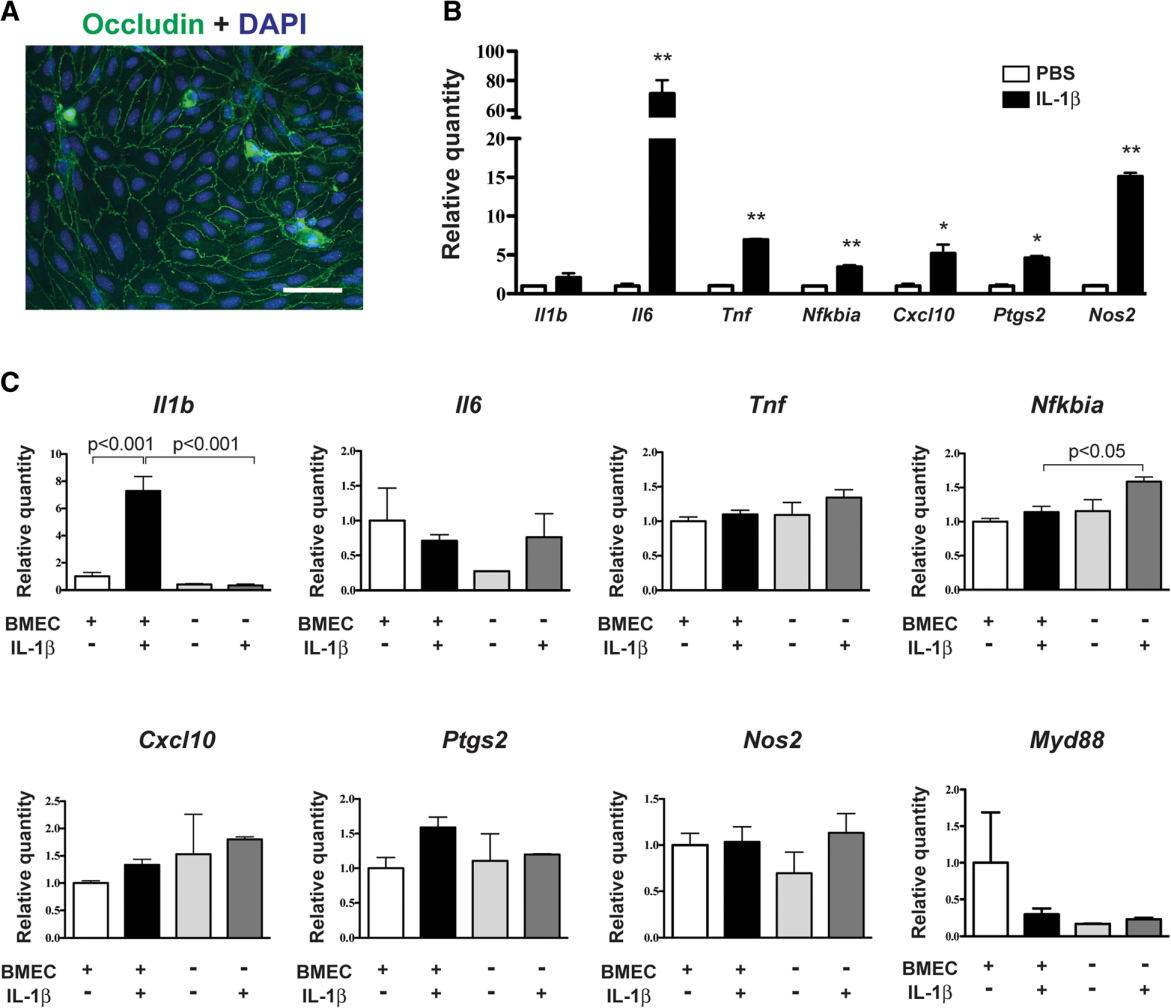
Co-cultured BMEC and SIM-A9 cells respond to luminal endothelial IL-1β treatment. **a** Occludin (*green*) immunofluorescence in cultured BMEC. *Blue* = DAPI staining. Scale bar *=* 50 μm. **b** Inflammatory gene expression in BMEC seeded into the upper chambers of transwell plates. PBS or IL-1β (50 ng/mL) was added to the upper chambers (i.e., the luminal surface of the BMEC) for 8 h. Data are representative of two independent experiments. *n* = 3 to *n* = 4 per group. Data are expressed as mean ± SEM. *p < 0.05, **p < 0.01 vs. PBS group. **c** Inflammatory gene expression in SIM-A9 cells seeded into the lower chambers of transwell plates containing BMEC in the upper chambers. PBS or IL-1β (50 ng/mL) was added to the upper chambers (i.e., the luminal surface of the BMEC) for 8 h. Wells of SIM-A9 cells without any BMEC in the upper inserts were also treated with PBS or IL-1β. *n* = 2 to *n* = 4 per group. Data are expressed as mean ± SEM

**Fig. 7:**
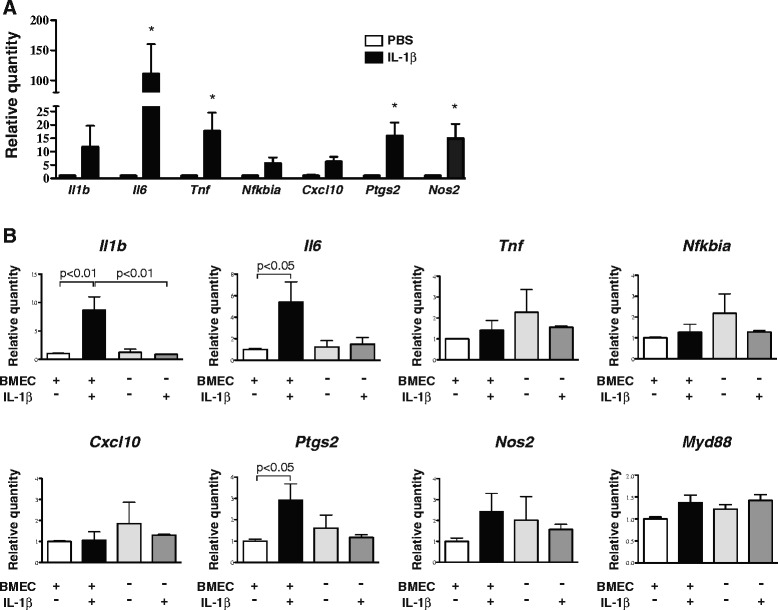
Co-cultured BMEC and SIM-A9 cells respond to abluminal endothelial IL-1β treatment. **a** Inflammatory gene expression in BMEC seeded into the upper chambers of transwell plates. PBS or IL-1β (50 ng/mL) was added to the lowers chambers (i.e., the abluminal endothelial surface) for 8 h. Data are representative of two independent experiments. *n* = 3 to *n* = 4 per group. Data are expressed as mean ± SEM. *p < 0.05 vs. PBS group. **b** Inflammatory gene expression in SIM-A9 cells seeded into the lower chambers of transwell plates containing BMEC in the upper chambers. PBS or IL-1β (50 ng/mL) was added to the lower chambers (i.e., the abluminal surface of the BMEC) for 8 h. Wells of SIM-A9 cells without any BMEC in the upper inserts were also treated with PBS or IL-1β. *n* = 2 to *n* = 3 per group. Data are expressed as mean ± SEM

Cultured BMEC establish a polarized barrier on transwell insert membranes, with the luminal (blood) endothelial surface facing the upper chamber and the abluminal (parenchymal) endothelial surface facing the lower chamber [30]. BMEC seeded onto transwell membranes form tight junctions, as demonstrated by occludin immunostaining (Fig. 6a). We first treated the upper chambers (i.e., the luminal endothelial surface) with PBS or IL-1β for 8 h. To control for any direct effects of IL-1β on the underlying SIM-A9 cells, we also included wells of SIM-A9 cells that contained empty inserts (i.e., no endothelial cells). With the exception of *Il1b* mRNA, all the other endothelial genes that we measured were upregulated by IL-1β treatment (Fig. 6b). The most pronounced increases were in *Il6* and *Nos2* mRNAs, which exhibited 71- and 15-fold increases, respectively. In the SIM-A9 cells in the lower chambers, *Il1b* was the only gene that was significantly upregulated (sevenfold) when IL-1β was added to the luminal endothelial surface (Fig. 6c). With the exception of a small (1.6-fold) yet statistically significant increase in *Nfkbia* mRNA, IL-1β-treatment did not alter gene expression in SIM-A9 cells that were cultured with empty transwell inserts. To evaluate whether SIM-A9 cells secrete mature IL-1β in response to endothelial-derived signals, we measured supernatant IL-1β levels in the lower chambers after 8 h of luminal BMEC treatment with PBS or IL-1β. We detected a small increase in IL-1β in supernatant from the wells that contained SIM-A9 cells compared to the wells with empty lower chambers, but the difference did not reach statistical significance (SIM-A9 + IL-1β: 113.6 ± 6 pg/mL vs. empty lower chambers + IL-1β: 103.7 ± 8 pg/mL, *p* > 0.05). In another experiment, we added IL-1β to the lower chambers (i.e., the abluminal endothelial surface) and observed patterns of endothelial gene expression that were similar to what we observed following luminal IL-1β exposure (Fig. 7a). In the underlying SIM-A9 cells, there was a ninefold induction of *Il1b* mRNA expression, and also significant increases in *Il6* (fivefold) and *Ptgs2* (threefold) gene expression in response to abluminal IL-1β treatment (Fig. 7b). In the absence of co-cultured endothelial cells, IL-1β application to the lower chambers did not alter SIM-A9 gene expression. Finally, we investigated whether nitric oxide signaling is an essential component of the microglial response to endothelial IL-1β treatment. Pre-treating co-cultured BMEC and SIM-A9 cells with the nitric oxide synthesis inhibitor l-NAME did not prevent luminal BMEC IL-1β treatment from inducing *Il1b* synthesis in SIM-A9 cells (Additional file 5).

## Discussion

IL-1β signaling in the CNS plays a critical role in innate immunity and cellular inflammatory responses. To address the question of which cells mediate IL-1β-induced disruptions in behavior, metabolism, and neuroendocrine function in vivo, many groups have genetically manipulated IL-1R1 or MyD88 expression in specific brain cell populations. For example, the Tie2 promoter is commonly exploited to target gene constructs to endothelial cells. We previously demonstrated that Tie2Cre-MyD88^Lox/Lox^ mice are completely resistant to anorexia, weight loss, fever, and reduced locomotor activity in response to icv IL-1β [8]. Using the Tie2 promoter to knockdown endothelial IL-1R1 expression [31] or to restore endothelial IL-1R1 expression in an IL-1R1 null background [32], other investigators have concluded that endothelial IL-1R1 signaling is necessary and/or sufficient for fever, reduced locomotor activity, CNS leukocyte infiltration, and activation of microglia and hypothalamic neurons in response to icv IL-1β. Although the Tie2 promoter is often described as an endothelial-specific Cre-driver, the Tie2 lineage is present in all microglia [33–35]. Thus, the relative contributions of endothelial vs. microglial IL-1β signaling in the generation of sickness responses cannot be distinguished in mice harboring genetic manipulations linked to the Tie2 promoter.

Endothelial cells are cellular targets for inflammatory mediators and play a role in generating tissue responses to systemic infections. In the non-inflamed brain, cerebrovascular cells are the principal sites of IL-1R1 expression [12], and disruption of IL-1β signaling in endothelium attenuates or abolishes IL-1β-induced fever and reduced locomotor activity [31, 36]. In the rodent brain, IL-1R1 mRNA and protein are expressed by parenchymal endothelial cells and perivascular cells in the choroid plexus and meninges [32, 37]. We demonstrated that IL-1β induces NF-κB nuclear localization in primary BMEC from WT, but not MyD88KO mice. This is consistent with reports that IL-1β rapidly induces NF-κB nuclear translocation and *Nfkbia* mRNA expression (a transcriptional marker of NF-κB activity) in the brain microvasculature following central or peripheral injection [38–40], and that this response is absent in MyD88-deficient mice [41]. We also observed that IL-1β elicits robust increases in mRNAs for inflammatory cytokines, adhesion molecules, the chemokine *Cxcl10*, and synthetic enzymes for production of nitric oxide and prostanoids in WT BMEC. Similar transcriptional and secretory profiles in response to IL-1β have also been demonstrated in BMEC in vivo [40] and in human and murine BMEC cell lines [42, 43].

In response to pathogens or tissue damage, microglia alter their morphology and release pro-inflammatory cytokines and chemokines. Microglia are activated by various inflammatory stimuli, including pathogen-associated molecular patterns (e.g., LPS) and cytokines (e.g., TNFα) [44, 45]. In vivo, microglia do not express IL-1R1 under basal conditions, although IL-1R1 is induced in hippocampal microglia following brain injury [13, 32]. Although we detected very low basal levels of *Il1r1* mRNA in our primary microglia cultures, we cannot rule out the possibility that this was due to *Il1r1* expressed by residual astrocytes (and/or other CNS cells) that were not removed during the trypsinization process. Similarly, Pinteaux et al. (2002) reported a low level of *Il1r1* mRNA expression in primary microglia cultures, but could not rule out oligodendrocyte progenitor cell contamination [46]. *Il1r1* mRNA was undetectable in SIM-A9 cells, nor was it detected in two other microglial cell lines [42]. Although microglia are a major source of IL-1β in the CNS, there is conflicting evidence in the literature regarding whether microglia themselves are direct targets for IL-1β signaling. IL-1β increased pro-inflammatory cytokine (*Il1b*, *Tnf*, and *Il6*) gene expression and chemokine (MIP-1α and MIP-1β) secretion in cultured human fetal microglia [47, 48]. In contrast, Pinteaux et al. (2002) did not observe changes in cytokine expression or secretion, NF-κB activation or MAPK activity in IL-1β-treated murine primary microglia [46]. Likewise, IL-1β did not induce inflammatory gene expression in the murine microglia cell lines EOC2 or EOC20 [42]. In our studies, IL-1β-treated primary microglia and SIM-A9 cells exhibited small increases in *Nfkbia* mRNA. The functional significance of this is unclear, because we did not detect NF-κB nuclear localization in isolated primary microglia or increased luciferase activity in NF-κB Luc SIM-A9 cells. We also observed small (1.5- to 2-fold) yet statistically significant increases in other inflammatory genes in primary microglia and SIM-A9 cells. The most pronounced effect of IL-1β was on *Nos2* gene expression, which was increased 7.8-fold in WT primary microglia (but was only marginally elevated at the same 4 h time point in SIM-A9 cells). *Nos2* is transcriptionally regulated by diverse inflammatory stimuli and is widely accepted as a marker for classical (M1) microglial activation [49, 50]. We conclude that isolated microglia do not exhibit significant inflammatory responses to IL-1β. However, it is possible that IL-1β pre-conditions microglia for the arrival of subsequent inflammatory stimuli (and potentially, a more robust inflammatory response) by shifting them toward an M1 phenotype.

Astrocytes express IL-1R1 [13, 46, 51] and are direct targets for IL-1β signaling in vitro [52]. We observed increased inflammatory gene expression in WT mixed glia and enriched astrocyte cultures in response to IL-1β, but not in corresponding cultures from MyD88KO mice. Furthermore, we observed abundant NF-κB nuclear localization in GFAP-positive astrocytes in IL-1β-treated WT mixed glia cultures. We next sought evidence of astrocyte-microglia crosstalk in response to IL-1β, similar to what other groups have demonstrated in response to LPS in vitro [23, 53]. We observed nuclear translocation of NF-κB in approximately 50% of microglia in IL-1β-treated mixed glia cultures. Because we did not observe IL-1β-induced nuclear NF-κB in isolated primary microglia or enhanced luciferase activity in isolated NF-κB Luc SIM-A9 cells, it is likely that astrocytes play an intermediary role in relaying the IL-1β signal to microglia. We observed increased luminescence in response to IL-1β when NF-κB Luc SIM-A9 cells were seeded into the same wells as WT mixed glia, but not when NF-κB Luc SIM-A9 cells were physically separated from IL-1β-treated WT mixed glia in transwells. These results are consistent with the hypothesis that direct physical contact between astrocytes and microglia is necessary for IL-1β-induced NF-κB activation in microglia, whereas soluble astrocyte-derived factors activate microglia via NF-κB-independent mechanisms.

We observed that conditioned media from IL-1β-treated astrocytes increased *Nos2* gene expression in primary microglia. However, *Nos2* gene expression was significantly reduced in SIM-A9 cells that were exposed to astrocyte-conditioned media, regardless of whether the astrocytes were treated with PBS or IL-1β, suggesting that astrocytes release signals that dampen microglial nitric oxide production. A role for astrocytes in inhibiting microglial Nos2 production has previously been demonstrated in LPS-treated mixed glia cultures [54]. The different *Nos2* responses of primary microglia and SIM-A9 cells could be indicative of a fundamental difference between the two cell types in their ability to respond to inflammatory signals. Alternatively, induction of *Nos2* mRNA could be due to residual astrocyte contamination in our primary microglia cultures.

We also investigated the possibility that IL-1β-treated brain endothelial cells relay inflammatory signals to microglia. When all three cell types (BMEC, astrocytes, and microglia) were cultured together in transwells, IL-1β induced NF-κB nuclear localization in approximately 50% of microglia. This percentage of microglial activation is comparable to what we observed in IL-1β-treated mixed glia cultures. IL-1β did not induce luciferase activity in NF-κB Luc SIM-A9 cells that were co-cultured with BMEC. In one of our transwell experiments, IL-1β-treated SIM-A9 cells exhibited greater induction of *Nfkbia* mRNA in the absence of BMEC than they did in the presence of BMEC, which suggests that endothelial cells release signals that dampen microglial NF-κB signaling. However, endothelial IL-1β treatment did increase inflammatory gene expression in SIM-A9 cells. When IL-1β was added to the luminal endothelial surface, only *Il1b* gene expression was induced in the underlying SIM-A9 cells. Adding IL-1β to the abluminal endothelial surface also induced *Il1b* mRNA expression in SIM-A9 cells, as well as *Il6* and *Ptgs2* mRNAs. The fact that SIM-A9 cells did not exhibit inflammatory responses to IL-1β in the absence of BMEC provides further confirmation that the SIM-A9 cells were not directly activated by IL-1β, but rather by an endothelial-derived signal. This is consistent with in vivo experiments demonstrating that after peripheral or central IL-1β injection, increased *Nfkbia* mRNA expression in brain endothelial cells precedes microglial activation [38, 40].

Given their polarization and position at the blood-brain interface, it is not surprising that brain endothelial cells have the ability to differentially respond to inflammatory signals arriving via the blood vs. locally generated within the CNS. IL-1R1 is expressed on both the luminal and abluminal endothelial cell membranes [55]. In our transwell experiments, IL-1β induced similar patterns of inflammatory gene expression in BMEC regardless of whether it was added to the luminal or abluminal endothelial membrane. BMEC can respond to inflammatory signals arriving on one surface and subsequently secrete cytokines from the opposing membrane [30]. Our finding that abluminal IL-1β treatment induced the expression of more diverse inflammatory genes in SIM-A9 cells than luminal IL-1β treatment is consistent with the observation that icv IL-1β injection elicits a broader profile of inflammatory gene expression in the brain (both in terms of number of genes and anatomical localization) than i.v. IL-1β treatment [40]. Compared to luminal LPS treatment, BMEC exhibit greater IL-6 secretion when LPS is applied to the abluminal surface [30]. It is possible that the abluminal endothelial surface is more responsive to inflammatory stimuli in general, perhaps due to different receptor expression profiles and/or densities on the luminal and abluminal membranes, membrane-specific activation of different intracellular signaling pathways, and/or crosstalk with adjacent inflammation-sensitive perivascular cells [56, 57].

Future studies will be devoted to identifying the astrocyte- and endothelial-derived signaling molecules that activate microglia under inflammatory conditions. Our observation that only astrocyte-derived signals induced NF-κB activity in NF-κB Luc SIM-A9 cells is consistent with the hypothesis that astrocytes and endothelial cells use different signaling mediators to activate microglia. Furthermore, it is plausible that BMEC release different signaling molecules depending upon which endothelial membrane is exposed to IL-1β, because the SIM-A9 transcriptional profile differed in response to luminal vs. abluminal IL-1β treatment. Based upon the BMEC transcriptional response to IL-1β, likely candidate molecules for endothelial-microglia communication include IL-6, TNFα, and prostaglandins. With respect to the latter, prostaglandin signaling is essential for IL-1β-induced fever [55, 58, 59], and pharmacological or genetic blockade of prostaglandin synthesis at least partly reverses IL-1β-induced anorexia [60, 61]. However, there are conflicting reports in the literature about whether brain endothelial or perivascular cells are the source of vascular prostaglandin production following IL-1β stimulation [55, 57, 62]. Nitric oxide is another possible mediator of endothelial-microglia signaling, because BMEC produce nitric oxide in response to inflammatory stimuli (including IL-1β), and treating primary microglia cultures with a nitric oxide donor increases IL-1β production [63, 64]. However, blocking nitric oxide production did not prevent luminal BMEC IL-1β treatment from inducing SIM-A9 *Il1b* synthesis. Finally, examining the role of direct communication between endothelial cells and astrocytes, as well as the involvement of other brain cell populations (e.g., neurons, pericytes, and oligodendrocytes) in propagating local inflammatory responses in the CNS is a worthy subject for future investigation.

## Conclusions

**Fig. 8:**
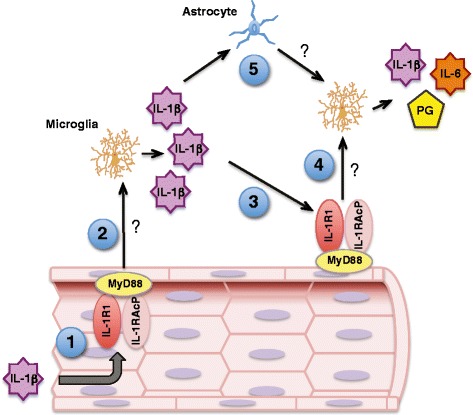
Relationship between brain endothelial and glial cells in initiating the brain’s inflammatory response to IL-1β. In the setting of systemic inflammation, *1*. circulating IL-1β binds to the IL-1R1 on the luminal surface of brain endothelial cells, *2*. Endothelial cells release signals into the brain parenchyma that increase microglial IL-1β production, *3*. Microglia-derived IL-1β engages IL-1R1 on the abluminal endothelial membrane, *4*. Triggering the release of signals that increase microglial production of IL-1β, IL-6, and prostaglandins, *5*. Astrocytes are also direct targets for microglia-derived IL-1β and activate microglia in a reciprocal fashion. *Question marks* indicate signaling molecules that have yet to be identified. This model explains how brain endothelial cells can differentially respond to inflammatory signals arising in the blood or brain and offers a mechanism by which brain endothelial and glial cells amplify, modify, and propagate inflammatory signaling within the CNS. *IL-1RAcP* IL-1R accessory protein, *PG* prostaglandins

We have generated a model that proposes how the microglial response to IL-1β likely depends upon an endothelial-derived intermediate, and thus does not require passage of IL-1β into the brain (Fig. 8). In response to systemic inflammation, circulating IL-1β engages IL-1R1 on the luminal endothelial surface, causing BMEC to secrete signals into the parenchyma that stimulate microglia to increase IL-1β synthesis and release. Microglial-derived IL-1β activates neighboring astrocytes, which produce paracrine signals that reciprocally activate microglia. Microglia-derived IL-1β also binds to IL-1R1 on the abluminal endothelial surface, causing BMEC to release signals that further increase microglial IL-1β production and also trigger IL-6 and prostaglandin synthesis. This sequential and reverberating cascade amplifies, modifies, and propagates inflammatory signaling within the CNS.

## Additional files

Additional file 1: Figure S1. Primary mixed glia and enriched astrocyte response to IL-1β is MyD88-dependent. Inflammatory gene expression in A. Primary mixed glia and B. Enriched astrocyte cultures from WT and MyD88KO mice that were treated with PBS or IL-1β (50 ng/mL) for 4 h. *n* = 4 per group. Data are expressed as mean ± SEM. *p < 0.05, **p < 0.01, ***p < 0.001 vs. WT PBS group, #*p* < 0.001 vs. WT IL-1β group. (PDF 474 kb)

Additional file 2: Figure S2. SIM-A9 cell transcriptional response to IL-1β. Inflammatory gene expression in SIM-A9 cells that were treated with PBS or IL-1β (50 ng/mL) for 1, 2, 4, 8, or 24 h. Gene expression in the IL-1β-treated cells is expressed as a percentage of the mean value for the PBS-treated cells at the same time point. *n* = 3 per group. Data are expressed as mean ± SEM. *p < 0.05, **p < 0.01 vs. PBS group at the same time point. (PDF 466 kb)

Additional file 3: Figure S3. Primary microglia and SIM-A9 cells respond to LPS and TNFα. Inflammatory gene expression in A. WT primary microglia and B. SIM-A9 cells that were treated with PBS or LPS (10 ng/mL) for 4 h. *n* = 4 per group. ***p < 0.001 vs. PBS-treated cells C. Inflammatory gene expression in SIM-A9 cells that were treated with PBS or TNFα (50 ng/mL) for 4 h. *n* = 4 per group. Data are expressed as mean ± SEM. *p < 0.05, ***p < 0.001 vs. PBS-treated cells. (PDF 468 kb)

Additional file 4: Figure S4. Lack of effect of astrocyte-conditioned media (CM) on SIM-A9 gene expression. A. Inflammatory gene expression in enriched astrocyte cultures that were treated with PBS or IL-1β (50 ng/mL) for 24 h. *n* = 4 per group. Data are expressed as mean ± SEM. **p < 0.01, ***p < 0.001 vs. PBS group. B. Inflammatory gene expression in SIM-A9 cells that were treated with PBS-treated astrocyte CM (PBS astrocyte CM) or IL-1β-treated astrocyte CM (IL-1β astrocyte CM) for 4 h. To control for direct effects of IL-1β on SIM-A9 cells, empty wells (containing no astrocytes) were treated with PBS or IL-1β for 24 h, and then the media was removed and applied to SIM-A9 cells for 4 h. These SIM-A9 groups are labeled as PBS and IL-1β, respectively. *n* = 3 to *n* = 4 per group. Data are expressed as mean ± SEM. ***p < 0.001 vs. PBS astrocyte CM group, #*p* < 0.05 vs. IL-1β astrocyte CM group, § vs. IL-1β group. (PDF 418 kb)

Additional file 5: Figure S5. Blockade of nitric oxide synthesis doesn’t alter SIM-A9 response to IL-1β-treated endothelial cells. A. Supernatant nitrite/nitrate levels, and B. SIM-A9 *Il1b* mRNA expression. BMEC were seeded into the upper inserts and SIM-A9 cells were seeded into the lower chambers of transwell plates. PBS or l-NAME (1 mM) was added to both the upper and lower chambers for 1 h, and then PBS or IL-1β (50 ng/mL) was added to the upper chambers for an additional 8 h. Nitrite/nitrate was measured in the supernatant from the upper chambers. *n* = 6 per group. Data are expressed as mean ± SEM. Bars with different superscripts are statistically different from one another (*p* < 0.05). (PDF 364 kb)

## Abbreviations

BMEC: Brain microvessel endothelial cells
BSA: Bovine serum albumin
CNS: Central nervous system
CXCL10: C-X-C motif chemokine 10
ICC: Immunocytochemistry
Icv: Intracerebroventricular
IL-1R1: Type I interleukin-1 receptor
IL-1β: Interleukin-1 beta
IL-6: Interleukin-6
l-LME: l-leucine methyl ester hydrochloride
l-NAME: Nω-Nitro-L-arginine methyl ester hydrochloride
LPS: Lipopolysaccharide
MyD88: Myeloid differentiation factor 88
MyD88KO: Myeloid differentiation factor 88 knockout
NF-κB: Nuclear factor kappa-light-chain-enhancer of activated B cells
TNFα: Tumor necrosis factor alpha
WT: Wild-type
