# Supplementary figures and images for: Amplification and propagation of interleukin-1β signaling by murine brain endothelial and glial cells
Source: J Neuroinflammation. 2017 Jul 1;14:133. doi: 10.1186/s12974-017-0908-4 (PMC5494131; doi:10.1186/s12974-017-0908-4)

**A**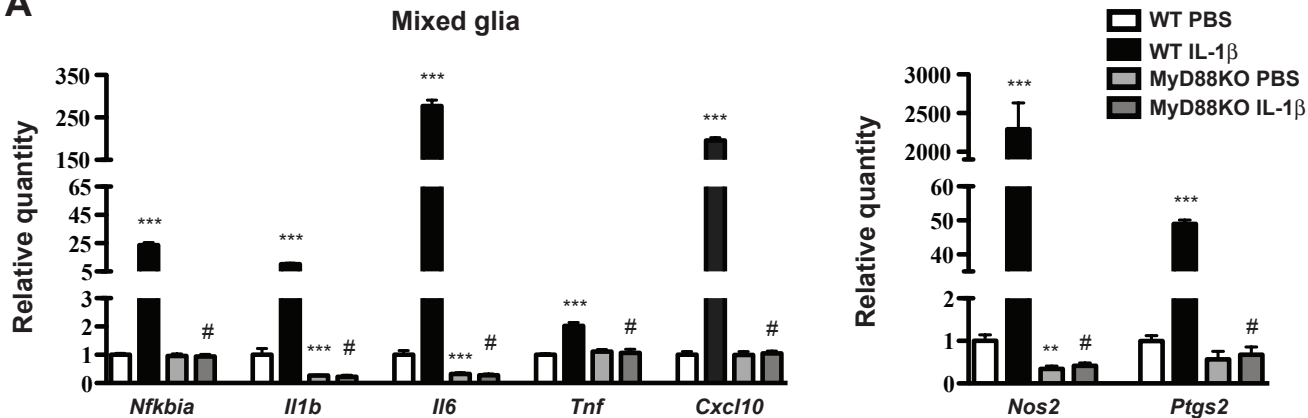**B**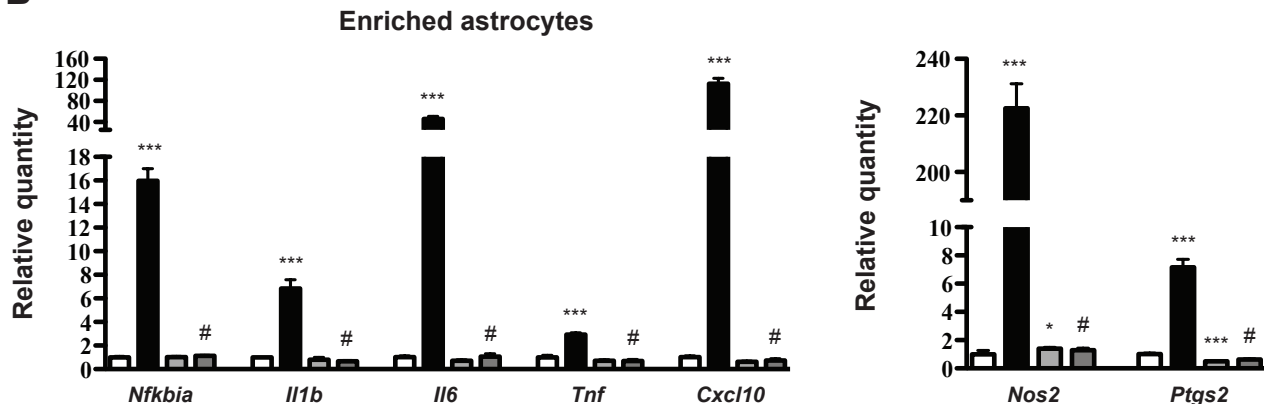

Supplement: Supplementary file 1 — Primary mixed glia and enriched astrocyte response to IL-1β is MyD88-dependent. Inflammatory gene expression in A. Primary mixed glia and B. Enriched astrocyte cultures from WT and MyD88KO mice that were treated with PBS or IL-1β (50 ng/mL) for 4 h. n = 4 per group. Data are expressed as mean ± SEM. *p < 0.05, **p < 0.01, ***p < 0.001 vs. WT PBS group, #p < 0.001 vs. WT IL-1β group. (PDF 474 kb) [file 12974_2017_908_MOESM1_ESM.pdf]

***Il1b***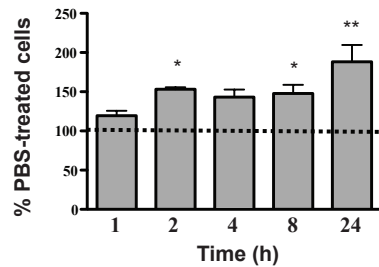***Il6***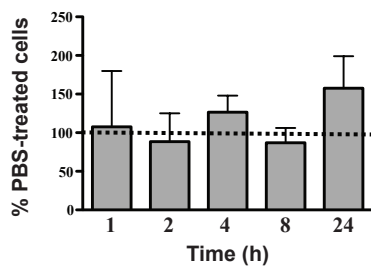***Tnf***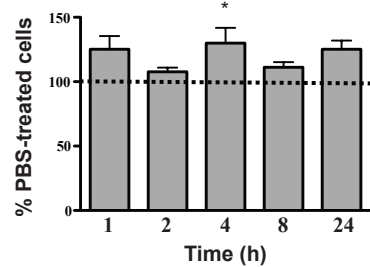***Nfkbia***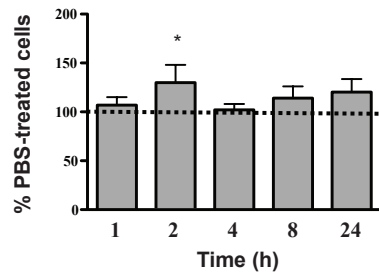***Cxcl10***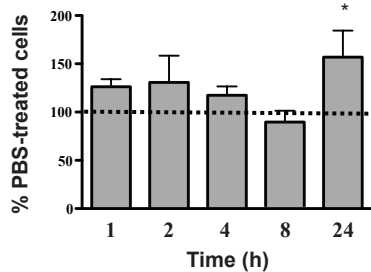***Nos2***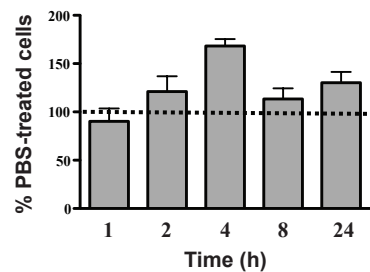***Ptgs2***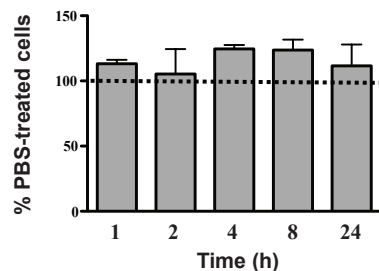

Supplement: Supplementary file 2 — SIM-A9 cell transcriptional response to IL-1β. Inflammatory gene expression in SIM-A9 cells that were treated with PBS or IL-1β (50 ng/mL) for 1, 2, 4, 8, or 24 h. Gene expression in the IL-1β-treated cells is expressed as a percentage of the mean value for the PBS-treated cells at the same time point. n = 3 per group. Data are expressed as mean ± SEM. *p < 0.05, **p < 0.01 vs. PBS group at the same time point. (PDF 466 kb) [file 12974_2017_908_MOESM2_ESM.pdf]

**A**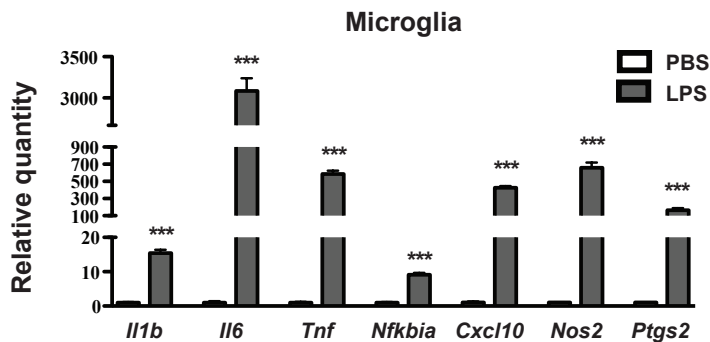**B**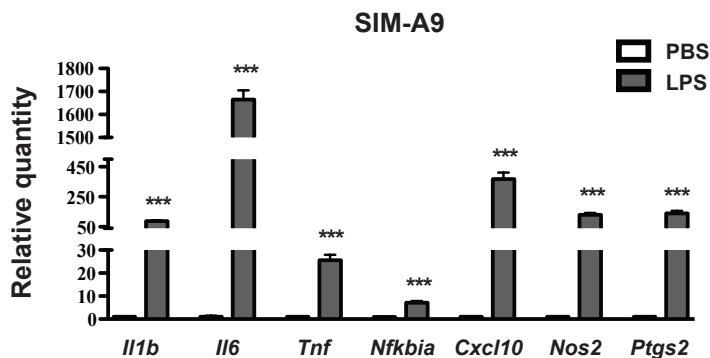**C**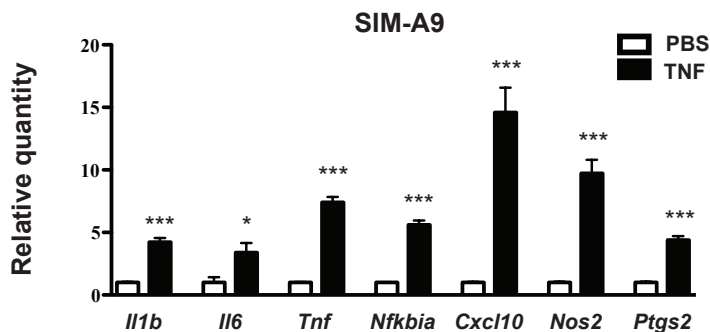

Supplement: Supplementary file 3 — Primary microglia and SIM-A9 cells respond to LPS and TNFα. Inflammatory gene expression in A. WT primary microglia and B. SIM-A9 cells that were treated with PBS or LPS (10 ng/mL) for 4 h. n = 4 per group. ***p < 0.001 vs. PBS-treated cells C. Inflammatory gene expression in SIM-A9 cells that were treated with PBS or TNFα (50 ng/mL) for 4 h. n = 4 per group. Data are expressed as mean ± SEM. *p < 0.05, ***p < 0.001 vs. PBS-treated cells. (PDF 468 kb) [file 12974_2017_908_MOESM3_ESM.pdf]

**A**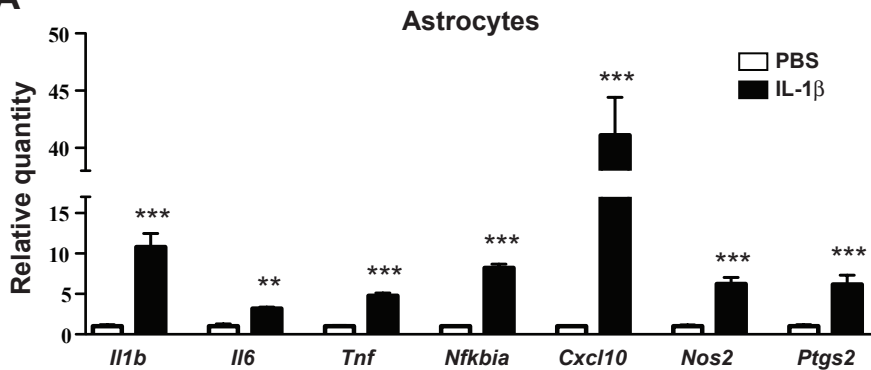**B**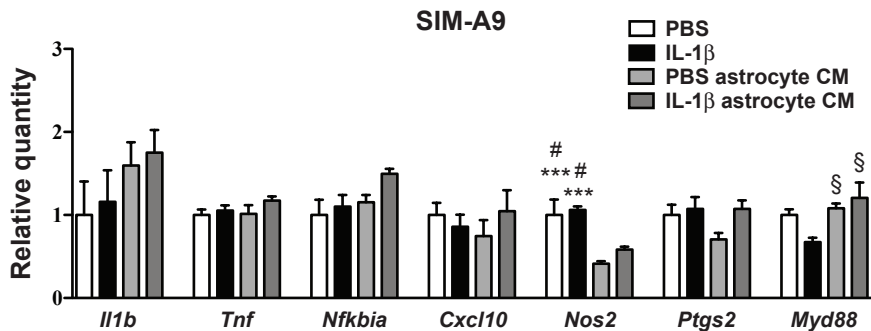

Supplement: Supplementary file 4 — Lack of effect of astrocyte-conditioned media (CM) on SIM-A9 gene expression. A. Inflammatory gene expression in enriched astrocyte cultures that were treated with PBS or IL-1β (50 ng/mL) for 24 h. n = 4 per group. Data are expressed as mean ± SEM. **p < 0.01, ***p < 0.001 vs. PBS group. B. Inflammatory gene expression in SIM-A9 cells that were treated with PBS-treated astrocyte CM (PBS astrocyte CM) or IL-1β-treated astrocyte CM (IL-1β astrocyte CM) for 4 h. To control for direct effects of IL-1β on SIM-A9 cells, empty wells (containing no astrocytes) were treated with PBS or IL-1β for 24 h, and then the media was removed and applied to SIM-A9 cells for 4 h. These SIM-A9 groups are labeled as PBS and IL-1β, respectively. n = 3 to n = 4 per group. Data are expressed as mean ± SEM. ***p < 0.001 vs. PBS astrocyte CM group, #p < 0.05 vs. IL-1β astrocyte CM group, § vs. IL-1β group. (PDF 418 kb) [file 12974_2017_908_MOESM4_ESM.pdf]

**A**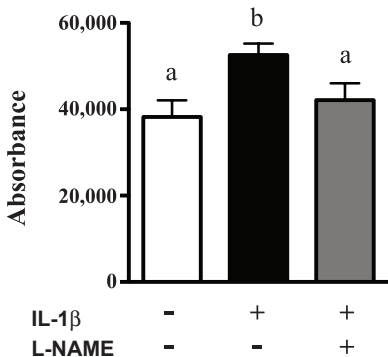**B**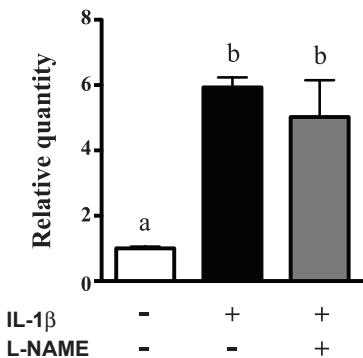

Supplement: Supplementary file 5 — Blockade of nitric oxide synthesis doesn’t alter SIM-A9 response to IL-1β-treated endothelial cells. A. Supernatant nitrite/nitrate levels, and B. SIM-A9 Il1b mRNA expression. BMEC were seeded into the upper inserts and SIM-A9 cells were seeded into the lower chambers of transwell plates. PBS or l-NAME (1 mM) was added to both the upper and lower chambers for 1 h, and then PBS or IL-1β (50 ng/mL) was added to the upper chambers for an additional 8 h. Nitrite/nitrate was measured in the supernatant from the upper chambers. n = 6 per group. Data are expressed as mean ± SEM. Bars with different superscripts are statistically different from one another (p < 0.05). (PDF 364 kb) [file 12974_2017_908_MOESM5_ESM.pdf]
